# Randomized controlled diagnostic trial to assess dRAST time to result and its utility for antimicrobial stewardship recommendation in bacteremic patients

**DOI:** 10.1128/spectrum.00288-25

**Published:** 2025-08-15

**Authors:** Ana Verónica Halperin, Rosa Escudero, María García-Castillo, María José Cardenas-Isasi, Francesca Gioia, Javier Cobo, Ana María Sánchez-Díaz, Rafael Cantón

**Affiliations:** 1 Servicio de Microbiología, Hospital Ramón y Cajal and Instituto Ramón y Cajal de Investigación Sanitaria (IRYCIS) 16507 , Madrid, Spain; 2 Servicio de Enfermedades Infecciosas, Hospital Ramón y Cajal and Instituto Ramón y Cajal de Investigación Sanitaria (IRYCIS) 537482, Madrid, Spain; 3 Service de Microbiología, Araba University Hospital, Osakidetza Basque Health Service, Vitoria-Gasteiz, Spain; 4 CIBER de Enfermedades Infecciosas (CIBERINFEC), Instituto Salud Carlos III (ISCIII) 637284 , Madrid, Spain; MultiCare Health System, Tacoma, Washington, USA

**Keywords:** antimicrobial stewardship, bacteremia, rapid tests, blood culture, rapid AST

## Abstract

Bloodstream infections cause great mortality and morbidity. Real-time microbiological information has been shown to have an impact on prognosis when combined with an antimicrobial stewardship (AMS) program. dRAST is a rapid commercial antimicrobial susceptibility testing (AST) system based on real-time microscopy. The primary outcome of this trial was to compare positive blood culture’s time-to-result (TTR), defined as time from Gram stain to AST report, between dRAST and our standard of care in a real-life clinical setting. Secondary outcomes included microbiological concordance, time to antimicrobial stewardship recommendation (TAMS), and time to change antimicrobial prescription (TCAP) in agreement with AMS recommendation. We performed a prospective, open-label, randomized (1:1) diagnostic clinical trial comparing dRAST and our standard of care commercial MicroScan broth microdilution (MBMD) (MicroScan-Walkaway, Beckman-Coulter, USA) in 277 hospitalized patients with bacteremia. Categorical and continuous variables were compared by chi-squared and Mann-Whitney *U* tests, respectively. Median TTR, TAMS, and TCAP were 25, 28, and 29 hours in the MBMD group, and 9, 11, and 13 hours in the dRAST group, respectively. For gram-negative bacilli, essential agreement (EA) was 93.6%, categorical agreement (CA) 93.2%, very major error (VME) 14%, major error (ME) 3%, and minor error (mE) 1.4%. For gram-positive cocci, EA was 93.7%, CA 92.8%, VME 0%, ME 7.6%, and mE 1.8%. dRAST is a reliable method for guiding an antimicrobial stewardship program for bacteremia, allowing significant reductions in TTR, TAMS, and TCAP that might impact patient management and clinical outcomes.

**IMPORTANCE:**

This study addresses a critical challenge in infectious diseases management by evaluating the efficacy of the dRAST system, a rapid antimicrobial susceptibility testing method, in guiding antimicrobial stewardship programs for patients with bloodstream infections. A randomized diagnostic trial was designed to address this objective. Our findings show that the use of dRAST significantly reduces time-to-result, time to antimicrobial stewardship recommendations, and time to appropriate therapy changes, particularly in critically ill patients. These results underline the potential of rapid diagnostic technologies to optimize patient outcomes and enhance antimicrobial stewardship efforts in real-world clinical settings.

## INTRODUCTION

Throughout the world, the number of patients at risk of bloodstream infections (BSIs) continues to rise (1, 2). BSIs are associated with high rates of morbidity and mortality ranging from 15% to 30% (3, 4), and they markedly increase the costs of hospital care. Blood culture remains the gold standard for the diagnosis of BSIs (5). The prompt identification of the causative agent(s) and the rapid initiation of appropriate antimicrobial therapy are critical for reducing mortality, especially in patients with septic shock (6–8). It has been consistently reported that rapid relay of microbiological findings is pivotal in patient management (9) and likely to improve survival in patients with BSI (10) in combination with an antimicrobial stewardship (AMS) program (11).

Inadequate empiric antimicrobial therapy is described in 20% of patients with BSI (12). This is most likely to happen in those infected with resistant pathogens, thus increasing mortality (13). Unsuspected antimicrobial resistance, in addition to delayed information of blood culture results, is associated with a higher mortality rate, especially for fast-growing bacteria such as Enterobacterales and *Staphylococcus aureus* (10, 11). To avoid this, broad-spectrum antimicrobials are recommended for patients with suspected severe infections to minimize the risk of undertreatment (14). On the other hand, the widespread use of broad-spectrum agents has been associated with negative consequences such as antimicrobial resistance and frequent adverse events, including allergic or hypersensitivity reactions, kidney injury, thrombocytopenia, *Clostridioides difficile* infection, and higher mortality (14–16). For these reasons, it is crucial to prescribe prompt, adequate treatment minimizing the impact of adverse effects by broader spectrum antimicrobials. The balance of these two might be achieved by faster, accurate antimicrobial susceptibility information in combination with an AMS program.

At the time of choosing appropriate empiric therapy, one should consider that delayed treatment increases mortality in patients with septic shock. Delays greater than 6 hours in septic patients without septic shock have been associated with higher mortality (17). In another retrospective study (18), delay in appropriate antimicrobial treatment was associated with mortality only if it was greater than 12 hours. These results provide an excellent time-window opportunity to offer useful rapid microbiological diagnostics, including antimicrobial susceptibility testing (AST), to guide initiation of therapy, provided there is a rapid blood culture positivity detection. Thus, successfully choosing appropriate treatment and limiting the unnecessary use of broad-spectrum antimicrobials. Since standard AST methods, like conventional broth microdilution (BMD), typically take longer than this timeframe (48–72 hours from blood culture positivity), rapid AST methods are demanded. The automated system dRAST (QuantaMatrix, Korea) allows rapid and direct AST using principles of microfluidics and automated imaging to provide results in 5–7 hours after blood culture positivity (19). This study aims to compare dRAST against our standard of care (SOC) AST method. The primary outcome was time-to-result (TTR), defined as the time from Gram stain to AST report. Secondary outcomes included microbiological concordance, time to antimicrobial stewardship recommendation (TAMS), and time to change antimicrobial prescription (TCAP).

## MATERIALS AND METHODS

We performed a single-center, prospective, open-label, randomized (1:1) diagnostic clinical trial comparing results obtained with dRAST (QuantaMatrix, Korea) and our standard of care commercial MicroScan broth microdilution (MBMD) (MicroScan-Walkaway, Beckman-Coulter, USA) from positive blood cultures of hospitalized patients with bacteremia over 18 years old. The inclusion period was from November 2021 to July 2023.

Positive blood cultures were processed as our SOC, until the point of microorganism identification when randomization was performed. After incubation in Bactec Plus Aerobic/F bottles (aerobic bottles) and Bactec Lytic/10 Anaerobic/F bottles in Bactec-FX (BD, USA), positive blood cultures bottles were Gram stained, subcultured in solid media, and—after a short incubation period (2–6 hours)—identification was performed by matrix-assisted laser desorption ionization time-of-flight mass spectrometry (MALDI-TOF) (Bruker, Germany) (20). Randomization was done after bacterial identification by sealed and numbered envelopes determined by aleatory assignment by our biostatistics department. For dRAST, either the aerobic or the anaerobic bottles can be used; we selected the one with earlier positivity for each case.

Exclusion criteria were positive blood cultures harboring microorganisms not included in dRAST panels (yeast, *Streptococcus* spp., gram-positive bacilli, gram-negative cocci, or anaerobic bacteria). In addition, *Staphylococcus aureus* was excluded due to the availability of *mecA* and *mecC* rapid detection system as part of our SOC (21), considering dRAST would not add any advantages for methicillin susceptibility reporting in our setting. Gram stains from positive blood cultures showing more than one microorganism morphology were assumed polymicrobial and excluded due to the impossibility to perform direct rapid MALDI-TOF identification and therefore dRAST inoculation. Patients with restricted treatment due to limited prognosis, previous participation in the study, or severe neutropenia (<500 neutrophils/µL) were also excluded, the latter being in agreement with our local guidelines that do not allow any de-escalation prior to AST report by microdilution.

Randomized samples assigned to dRAST were processed according to the manufacturer’s instructions with software version V.1.5.010. All cases assigned to the dRAST arm were also analyzed in parallel with MBMD to determine AST agreement. Resistance mechanisms were detected phenotypically following EUCAST recommendations (version 2.01, July 2017). If carbapenem resistance was observed, carbapenemase detection was performed either by immunochromatography with OKNVI RESIST-5 (Coris BioConcept, Gembloux, Belgium) or PCR with Xpert Carba-R assay (Cepheid, Sunnyvale, CA, USA). Categorical agreement (CA) and essential agreement (EA) were measured as recommended previously (22). Minimal inhibitory concentrations (MIC) were interpreted according to EUCAST v13.1 (23).

All AST results (dRAST and MBMD) were uploaded in real time in the patient’s electronic medical record and communicated by phone to the infectious disease specialist participating in the AMS team. Based on AST results, patients in both arms received an AMS recommendation for targeted antimicrobial treatment: to maintain the same antimicrobial, to change it (add a new antimicrobial, narrow spectrum, broaden spectrum, or suspend antimicrobial therapy), to change the dose, or to switch to oral treatment. Only written recommendations in the electronic medical record were considered for this study. All time and clinical values were extracted from the hospital’s information system and collected in REDCap (13.14.11).

TTR was defined as the time from Gram stain to AST report. TAMS and TCAP were also calculated from the time of Gram stain. When the AMS recommendation was to maintain the same treatment, TCAP was not calculated as there were no changes in treatment expected. Twenty-seven patients (9.7%) who did not receive a written AMS recommendation were not evaluated for TAMS or TCAP.

Data were analyzed with STATA (version 16.0). Sample size was calculated based on a potency of (1 − *β*) =80% and an error of 5% (*n* = 286). Categorical and continuous variables were compared by chi-squared and Mann-Whitney *U* tests, respectively.

## RESULTS

**Fig 1:**
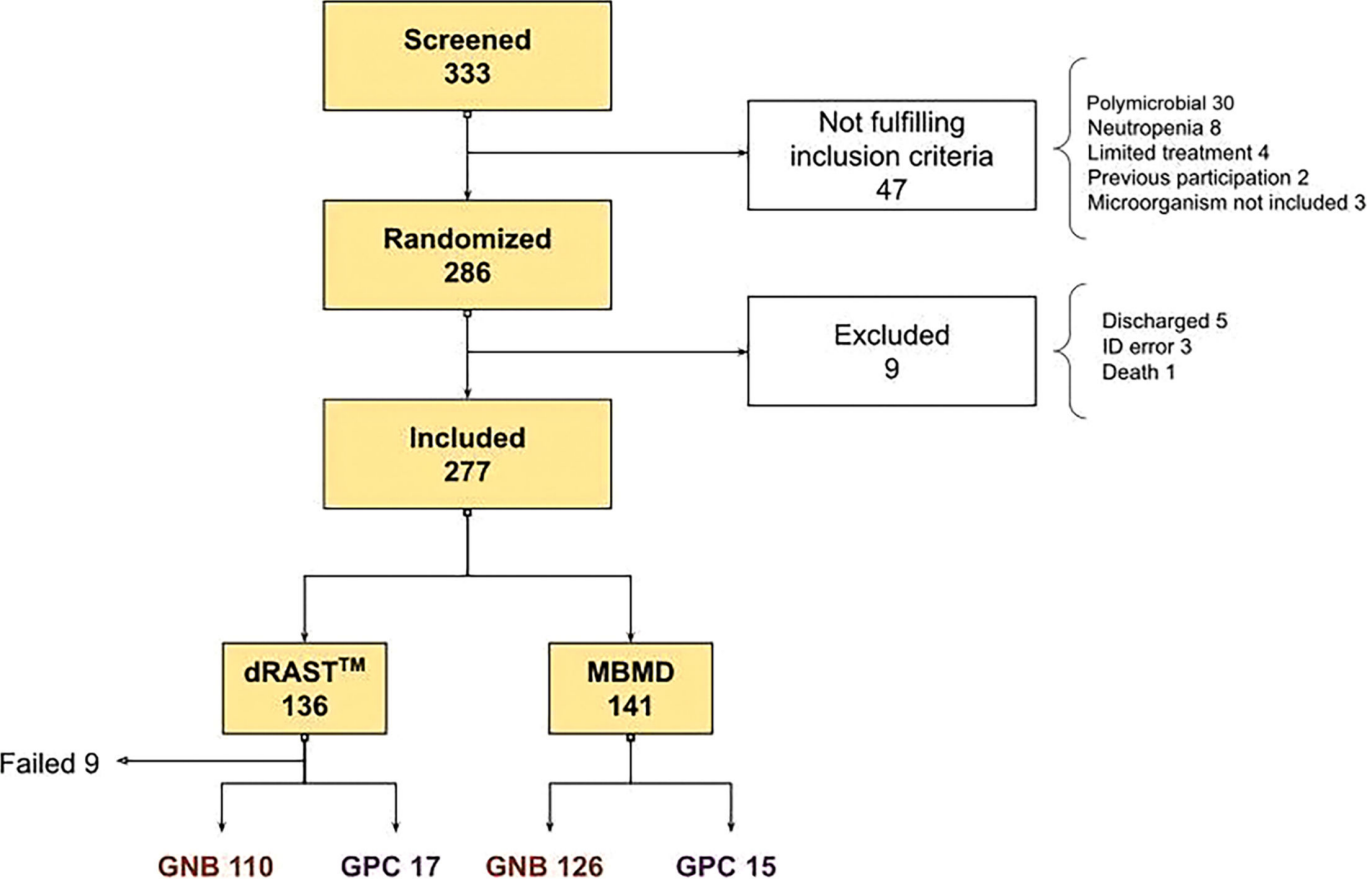
Patient inclusion flowchart. Of 333 screened patients, we included a total of 277. From the 136 patients included in the dRAST arm, nine panels had a mechanical error in the system (eight GNB and one GPC). GNB, gram-negative bacilli; GPC, gram-positive cocci.

We screened 333 patients with positive blood cultures, of whom 286 fulfilled the inclusion criteria. Out of these, nine patients were excluded after randomization: five were discharged before any blood cultures were positive, one subject died before any blood cultures were flagged positive, and three samples were found to have labeling errors in the blood culture vials. Finally, 277 positive blood cultures were included, 141 in the MBMD arm, and 136 in the dRAST arm. Figure 1 shows the patients’ inclusion flow chart. Baseline characteristics are shown in Table 1. The median age was 76 years (interquartile range [IQR] 65–85), 51.6% of the patients were male, with a homogeneous distribution in both groups. Comorbidities were very similar in both groups, shown by a median Charlson index of 2 points. Sepsis criteria were met by 31% of the subjects, defined by Sepsis-3 criteria (24). The most frequent source of bloodstream infection was the urinary tract, being over 50% for both groups, followed by abdominal infection (27%). Forty-five percent of the infections were nosocomial or healthcare-related. Thirty percent of all patients had received antimicrobial treatment before blood culture sampling.

**TABLE 1:** Baseline characteristics^*c*^

	Total (277)		MBMD (141)		dRAST (136)		*P*
Male	143	51.60%	76	53.90%	67	49.30%	0.44
Age (median, IQR) (y)	76 (65–85)		76 (65–86)		78 (64.5–87)		0.89
Ward							
Medical	201	72.60%	95	67.40%	106	77.90%	0.05
Surgical	50	18.10%	29	20.60%	21	15.40%	
ICU	24	8.70%	16	11.30%	8	5.90%	
ER	2	0.72%	1	0.70%	1	0.7	
Sepsis	86	31%	44	31.20%	42	30.90%	0.95
Comorbidities							
Charlson (median, IQR)	2 (1–3)		2 (1–3)		2 (1–3)		0.8
Immunosuppression^*a*^	63	22.70%	33	23.40%	30	22.10%	0.79
Microorganism							
GPC	33	11.90%	15	10.60%	18	13.20%	0.28
*E. faecalis*	14	5.10%	8	5.70%	6	4.40%	
*E. faecium*	10	3.60%	2	1.40%	8	5.90%	
CoNS	9	3.30%	5	3.50%	4	2.90%	
GNB	244	88.10%	126	89.40%	118	86.80%	0.89
*E. coli*	160	57.80%	83	58.90%	77	56.60%	
*Klebsiella* spp.	49	17.70%	25	17.70%	24	17.60%	
*Enterobacter* spp.	4	1.40%	2	1.40%	2	1.50%	
*Citrobacter* spp.	4	1.40%	2	1.40%	2	1.50%	
*Serratia* spp.	2	0.70%	2	1.40%	0	0%	
*Providencia* spp.	1	0.40%	0	0%	1	0.70%	
*Morganella* spp.	1	0.40%	1	0.70%	0	0%	
*Proteus* spp.	8	2.90%	4	2.80%	4	2.90%	
*Pseudomonas aeruginosa*	6	2.20%	3	2.10%	3	2.20%	
*Acinetobacter* spp.	1	0.40%	1	0.70%	0	0%	
*Stenotrophomonas maltophilia*	2	0.70%	1	0.70%	1	0.70%	
Others	6	2.20%	2	1.40%	4	2.90%	
Resistance mechanism	47	17.00%	25	17.70%	22	16.20%	0.73
ESBL	32	11.60%	16	11.30%	16	11.80%	
AmpC^*b*^	3	1.10%	3	2.10%	0	0.00%	
CPE	8	2.90%	5	3.50%	3	2.20%	
GRE	2	0.70%	1	0.70%	1	0.70%	

^*a*^ Immunosuppression is defined as at least one of the following: HIV clinical category C, active chemotherapy, neutrophil count <1,000/µL, biologic therapy or other immunosuppressive drugs, chronic corticosteroid treatment, solid organ transplant, and hematopoietic stem cell transplantation.

^*b*^ Chromosomal hyperproduced AmpC.

^*c*^ ICU, intensive care unit; y, years; ER, emergency room; GPC, gram-positive cocci; GNB, gram-negative bacilli; CoNS, coagulase-negative *Staphylococcus* spp.; ESBL, extended-spectrum beta lactamase; CPE, carbapenemase-producing Enterobacterales; GRE, glycopeptide-resistant *Enterococcus*.

Of all the bacterial isolates grown in blood cultures, 244 were gram-negative bacilli (GNB) and 33 were gram-positive cocci (GPC). A summary of species and resistance mechanisms detected is presented in Table 1.

Microbiological agreement for each antibiotic tested is presented in Tables 2 and 3. Detailed information about each antibiotic can be found in the supplementary tables. Nine of the dRAST panels had a mechanical error in the system (eight GNB and one GPC); therefore, their agreement with MBMD could not be evaluated, but they were included in the final analysis as intended to treat. For GNB, EA was 93.6%, CA 93.2%, very major error (VME) 14.0%, major error (ME) 3%, and minor error (mE) 1.4%. VMEs were mainly due to piperacillin-tazobactam (21.4%), gentamicin (41.2%), and amikacin (100%), although other antibiotics have also shown VME and ME above 3%. For GPC, EA was 93.7%, CA 92.8%, VME 0%, ME 7.6% and mE 1.8%.

**TABLE 2:** Microbiological agreement for gram-negative bacilli^*a*^

	AMP	PIP	AMC	PTZ	CTX	CAZ	CEF	IMI	MER	CIP	LVX	GEN	AMK	COL
*n*	107	105	106	109	107	109	110	109	109	110	109	106	109	107
*R*	77	64	28	14	22	20	19	2	2	30	28	17	6	4
EA (%)	86.8	86.5	95.3	96.3	93.4	92.7	93.6	96.3	92.7	95.5	96.3	92.5	94.5	98.1
CA (%)	93.5	84.8	83.3	94.5	93.5	90.8	93.6	92.7	97.2	94.5	93.6	92.5	94.5	99.1
VME	7 (9.1%)	9 (14.1%)	3 (10.7%)	3 (21.4%)	4 (18.2%)	2 (10%)	2 (10.5%)	0	0	3 (10%)	3 (10.7%)	7 (41.2%)	6 (100%)	0
ME	0	7 (17.1%)	14 (18.9%)	2 (2.2%)	1 (1.2%)	1 (1.2%)	2 (2.3%)	3 (2.9%)	1 (0.9%)	1 (1.3%)	3 (3.8%)	1 (1.1%)	0	1 (1%)
mE	0	0	0	1 (0.9%)	1 (1.9%)	7 (6.4%)	3 (2.8%)	5 (4.6%)	2 (1.8%)	2 (1.8%)	1 (0.9%)	0	0	0

^*a*^ *n*, number of total isolates tested; *R*, number of resistant isolates tested; AMP, ampicillin; PIP, piperacillin; AMC, amoxicillin-clavulanate; PTZ, piperacillin-tazobactam; CTX, cefotaxime; CAZ, ceftazidime; CEF, cefepime; IMI, imipenem; MER, meropenem; CIP, ciprofloxacin; LVX, levofloxacin; GEN, gentamicin; AMK, amikacin; COL, colistin.

**TABLE 3:** Microbiological agreement for gram-positive cocci^*a*^

	AMP	OXA	ERI	CLI	VAN	TEI	DAP	LNZ	GEN	LVX	RIF	TET	FUS
*n*	13	4	4	4	17	17	4	17	4	17	3	4	3
*R*	8	3	3	1	1	1	0	1	1	11	0	0	1
EA (%)	100	75	100	100	100	100	100	70.6	100	94.1	100	100	100
CA (%)	100	75	100	75	100	100	100	82.4	100	82.4	100	100	100
VME	0	0	0	0	0	0	–	0	0	0	–	–	0
ME	0	1 (100%)	0	0	0	0	0	3 (18.8%)	0	2 (40%)	0	0	0
mE	0	0	0	1 (25%)	0	0	0	0	0	1 (5.9%)	0	0	0

^*a*^ *n*, number of total isolates tested; *R*, number of resistant isolates tested; AMP, ampicillin; OXA, oxacillin; ERI, erythromycin; CLI, clindamycin; VAN, vancomycin; TEI, teicoplanin; DAP, daptomycin; LZD, linezolid; GEN, gentamicin; LVX, levofloxacin; RIF, rifampin; TET, tetracycline; FUS, fusidic acid. –, VME could not be calculated due to absence of resistant isolates.

Of all the included subjects, 277 (97.5% in total, 136 and 141 in MBMD and dRAST, respectively) had empiric antimicrobial treatment at the time of the diagnosis of bacteremia. There were 27 patients (9.7%) who did not receive a written AMS recommendation (22 in MBMD and 5 in dRAST) and therefore could not be evaluated for TAMS or TCAP. Overall, the most common AMS recommendations were to maintain the same treatment (37.2%) or to narrow the antimicrobial spectrum (35.7%) (Table 4). However, in septic subjects, the most frequent AMS recommendation was to narrow the antimicrobial spectrum (71% and 79% for MBMD and dRAST, respectively).

**TABLE 4:** Summary of antimicrobial stewardship recommendations^*a*^

	Total (277)	MBMD (141)	dRAST (136)
Maintain ATB	103 (37.2%)	45 (31.9%)	58 (42.6%)
Change ATB	140 (50.5%)	71 (50.4%)	69 (50.7%)
Add new ATB	21 (7.6%)	11 (7.8%)	10 (7.4%)
Narrow ATB spectrum	99 (35.7%)	51 (36.2%)	48 (35.3%)
Broaden ATB spectrum	16 (5.8%)	7 (5%)	9 (6.6%)
Suspend ATB	4 (1.4%)	2 (1.4%)	2 (1.5%)
Change dose	4 (1.4%)	1 (0.7%)	3 (2.2%)
Change to oral administration	8 (2.9%)	4 (2.8%)	4 (2.9%)
No written recommendation	27 (9.7%)	22 (15.6%)	5 (3.7%)

^*a*^ ATB, antimicrobial. Some patients received more than one recommendation (i.e., narrow spectrum and change to oral administration).

**Fig 2:**
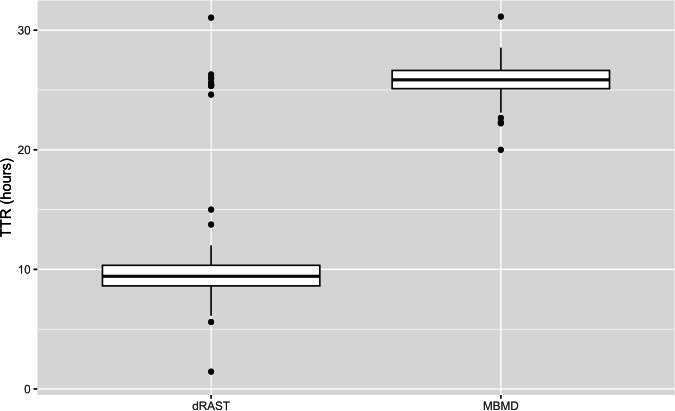
Median TTR. Boxplot representing the primary outcome, time to result (TTR) in hours, defined as the time from Gram stain report of positive blood cultures to the time of AST report. Median TTR for MBMD was 25 hours, median TTR for dRAST was 9 hours, and *P* < 0.001.

Compliance with AMS recommendations was 77.3% for MBMD and 83.1% for dRAST. Median TTR, TAMS, and TCAP were 25, 28, and 29 hours in the MBMD group, and 9, 11, and 13 hours in the dRAST group, respectively (Table 5). Overall, median TTR was reduced by 16 hours in the dRAST group (Fig. 2), while median TAMS and TCAP were reduced by 17 hours and 16 hours, respectively (*P* < 0.001). When considering septic patients only, median TAMS and TCAP were reduced by 18 and 28 hours, respectively.

**TABLE 5:** Overview of time endpoints^*a*^

	Total (277)		MBMD (141)		dRAST (136)		*P*
	Mean ± SD	Median (IQR)	Mean ± SD	Median (IQR)	Mean ± SD	Median (IQR)	
TTR: Gram to AST	20 ± 12.6	23.8 (9–25.9)	29 ± 10	25 (25–26)	10.9 ± 5.8	9 (8.6–10.4)	<0.001
TAMS	31 ± 27	25 (10–38)	45 ± 29	28 (25–58)	18 ± 19	11 (9–15)	<0.001
TCAP	35.9 ± 32	26 (11–50)	46 ± 34.8	29 (25–53)	25 ± 24	13 (10–26)	<0.001
T-Gram to end ATB	300 ± 227	228 (169–337)	303 ± 249	220 (169–324)	296 ± 202	228 (169–360)	0.47

^*a*^ TTR, time-to-result defined as time in hours since Gram stain report to AST report; TAMS, time to antimicrobial stewardship recommendation in hours; TCAP, time to change antimicrobial prescription in hours; T-Gram to end ATB, time in hours since Gram report to end of antimicrobial treatment.

There were no significant differences in 30-day mortality, total length of stay (LOS), or *Clostridioides difficile* infection between both groups (Table 6); however, median intensive care unit (ICU) LOS was reduced by 50%, from 8 (IQR: 5–30) to 4 days (IQR: 2–6.5) (tendency not statistically significant, *P* = 0.14).

**TABLE 6:** Clinical outcomes^*a*^

	Total (277)		MBMD (141)		dRAST (136)		*P*
30-day mortality	32	11.6%	19	13.5%	13	9.6%	0.07
Multi-resistant reinfection	12	4.3%	7	5.0%	5	3.7%	0.69
Re-admission	22	7.9%	9	6.4%	13	9.6%	0.5
CDI	7	2.5%	3	2.1%	4	2.9%	0.67
Stay since Gram (d), median (IQR)	7.5 (4.5–13.5)		7.5 (4.5–12.5)		7.5 (3.5–14.4)		0.68
Total length of stay (d), median (IQR)	10 (6–18)		10 (6–18)		10 (6–19)		0.68
ICU admission	33	11.9%	17	12.1%	16	11.8%	0.94
ICU duration (d), median (IQR)	6 (2–12)		8 (5–30)		4 (2–6.5)		0.14

^*a*^ CDI, *Clostridioides difficile* infection; d, days.

## DISCUSSION

We have performed a real-life, single-center randomized diagnostic trial, which showed that dRAST allows quick AST results with EA and CA that are above 90% when performed in a real-life scenario. These results are in accordance with previous publications (25). However, when antimicrobials were analyzed individually, VME rates were above the recommended value (>3%) for ampicillin, piperacillin, amoxicillin-clavulanate, piperacillin-tazobactam, cefotaxime, ceftazidime, cefepime, ciprofloxacin, levofloxacin, gentamicin, and amikacin. ME was also above 3% for piperacillin and amoxicillin-clavulanate. This could be explained by a number of reasons: firstly, the software version used was updated after the study was finished, and this could bring an improvement in the future that could reduce errors, as newer versions of microscopic imaging processing might retrieve better results in the future. Secondly, a higher proportion of VME and ME might also be due to MIC value variations, a fact recognized by EUCAST with the inclusion of an area of technical uncertainty in their breakpoint tables (23). Additionally, piperacillin-tazobactam, in particular, has been associated with many difficulties in validation in the past (26, 27). Finally, the number of resistant isolates tested was low for amoxicillin-clavulanate, piperacillin-tazobactam, cephalosporins, quinolones, and aminoglycosides, therefore resulting in a larger proportion of VME.

The results obtained by dRAST in combination with our AMS program allowed us to significantly reduce the median TTR and TCAP therapy by 16 hours. We saw an even greater reduction (28 hours) in median TCAP for the subgroup of septic patients in the dRAST arm. This might be due to the initial broader spectrum of antimicrobials used for empiric treatment in patients who present more severely ill, as shown by a greater proportion of de-escalation once AST is available.

Regarding clinical outcomes, 30-day mortality showed a decreased tendency for the dRAST group (*n* = 19 vs 13), although the sample size is small and it did not reach statistical significance. Overall, the number of deaths was low in our study; a larger sample would be required to evaluate this event. Nevertheless, rapid AST has been shown to have clear benefits. In a previous meta-analysis (11), rapid AST in combination with an AMS program proved to have a positive effect on reducing mortality, especially for GNB. Similarly, LOS did not show any significant changes either. However, when only analyzing critically ill patients admitted to ICU, there was a 50% reduction in LOS, from 8 to 4 days (tendency, not statistically significant). This reduction of LOS might impact the cost of patients’ care, as demonstrated in other studies (28).

Our study has several limitations. Firstly, some microorganisms not suitable for dRAST panels, as well as polymicrobial blood cultures, could not be included. For these reasons, our conclusions could only potentially be applied mainly to Enterobacterales and enterococcal bacteremia. *S. aureus* was excluded due to the availability of a rapid genetic test for methicillin resistance in our center (21). A wide number of additional rapid tests are also available to detect resistance mechanisms in other bacteria, especially GNB (i.e., extended-spectrum beta lactamase and carbapenemase lateral flow-immunoassay and carbapenemase gene detection). However, the resistance phenotype can be less predictable in GNB, whereas methicillin resistance can be predicted more easily in *S. aureus* by the detection of *mecA* and *mecC* genes. For multi-resistant GNB, dRAST could have the advantage of providing a more complete and phenotypic antibiogram that also reports MICs. This includes resistance to beta-lactams and other antimicrobials determined by genes not included in the other available rapid kits, or that are the results of the combination of multiple mechanisms. dRAST could have the advantage in comparison to other rapid AST methods of being mostly automatic with little hands-on time and reporting both MICs and interpretation of AST.

Another limitation is that our study was performed in a single site, and although the number of patients included was large, the results might not be applicable in other centers with different populations, resources, or a different AMS program. Lastly, we compared the microbiological results to our standard of care (automatized microdilution in Microscan-Walkaway), and not to the reference BMD (ISO 20776-2:2019); therefore, microbiological agreement can only be assessed in this context. However, the method has been previously validated with an overall concordance of over 90% (25).

In conclusion, dRAST can provide a powerful tool to support faster AMS decision making for targeted antimicrobial therapy in patients with bacteremia, and it could be particularly useful in septic patients and critically ill patients admitted to the ICU.

## SUPPLEMENTAL MATERIAL

Supplemental tables spectrum.00288-25-s0001.docx Tables S1 and S2.

ASM does not own the copyrights to Supplemental Material that may be linked to, or accessed through, an article. The authors have granted ASM a non-exclusive, world-wide license to publish the Supplemental Material files. Please contact the corresponding author directly for reuse.
