# Supplementary material for: Randomized controlled diagnostic trial to assess dRAST time to result and its utility for antimicrobial stewardship recommendation in bacteremic patients
Source: Microbiol Spectr. 2025 Aug 15;13(10):e00288-25. doi: 10.1128/spectrum.00288-25 (PMC12502579; doi:10.1128/spectrum.00288-25)
Supplement: Supplemental tables — Tables S1 and S2. [file spectrum.00288-25-s0001.docx]

**Supplementary Table 1: Microbiological agreement for Gram negative bacilli**

|  | **AMP** | **PIP** | **AMC** | **PTZ** | **CTX** | **CAZ** | **CEF** | **IMI** | **MER** | **CIP** | **LVX** | **GEN** | **AMK** | **COL** | **STX** |
| --- | --- | --- | --- | --- | --- | --- | --- | --- | --- | --- | --- | --- | --- | --- | --- |
| ***E. coli*** (n=73) | | | | | | | | | | | | | | | |
| EA (%) | 86.1 | 87.5 | 94.4 | 95.9 | 95.8 | 94.5 | 94.5 | 100 | 98.6 | 94.5 | 97.3 | 91.8 | 94.5 | 100 | NT |
| CA (%) | 90.4 | 86.3 | 78.9 | 94.5 | 94.5 | 91.8 | 94.5 | 100 | 100 | 93.2 | 94.5 | 91.4 | 94.5 | 100 | NT |
| VME | 7(15.2%) | 8(18.2%) | 2(12.5%) | 2(33.3%) | 1(8.3%) | 1(9.1%) | 2(16.7%) | - | - | 2(9.5%) | 2(9.5%) | 5(41.7%) | 4(100%) | - | NT |
| ME | 0 | 2(6.9%) | 13(23.6%) | 2(3%) | 1(1.7%) | 1(1.7%) | 1(1.6%) | 0 | 0 | 1(2%) | 1(2%) | 1(1.6%) | 0 | 0 | NT |
| mE | 0 | 0 | 0 | 0 | 2(2.7%) | 4(5.5%) | 1(1.45%) | 0 | 0 | 2(2.7%) | 1(1.4%) | 0 | 0 | 0 | NT |
| ***K. pneumoniae*** (n=18) | | | | | | | | | | | | | | | |
| EA (%) | 100 | 83.3 | 100 | 94.4 | 94.4 | 100 | 94.4 | 94.4 | 94.4 | 100 | 94.4 | 94.4 | 88.9 | 94.4 | NT |
| CA (%) | 100 | 77.8 | 94.1 | 88.9 | 94.4 | 94.4 | 94.4 | 88.9 | 94.4 | 100 | 88.9 | 94.4 | 88.9 | 94.4 | NT |
| VME | 0 | 0 | 1(12.5%) | 1(14.3%) | 1(14.3%) | 0 | 0 | 0 | 0 | 0 | 0 | 1(33.3%) | 2 100%) | 0 | NT |
| ME | 0 | 4(100%) | 0 | 0 | 0 | 0 | 0 | 0 | 0 | 0 | 2(14.3%) | 0 | 0 | 1(5.6%) | NT |
| mE | 0 | 0 | 0 | 1(5.6%) | 0 | 1(5.6%) | 1(5.6%) | 2(11.1%) | 1(5.6%) | 0 | 0 | 0 | 0 | 0 | NT |
| ***K. oxytoca*** (n=2) | | | | | | | | | | | | | | | |
| EA (%) | 50 | 50 | 100 | 100 | 50 | 50 | 100 | 100 | 100 | 100 | 100 | 100 | 100 | 100 | NT |
| CA (%) | 100 | 100 | 50 | 100 | 50 | 50 | 100 | 100 | 100 | 100 | 100 | 100 | 100 | 100 | NT |
| VME | 0 | 0 | - | 0 | 1(100%) | - | - | - | - | - | - | - | - | - | NT |
| ME | 0 | - | 1 (50%) | 0 | 0 | 0 | 0 | 0 | 0 | 0 | 0 | 0 | 0 | 0 | NT |
| mE | 0 | 0 | 0 | 0 | 0 | 1(50%) | 0 | 0 | 0 | 0 | 0 | 0 | 0 | 0 | NT |
| ***K. aerogenes*** (n=2) | | | | | | | | | | | | | | | |
| EA (%) | 100 | 100 | 100 | 100 | 100 | 100 | 100 | 100 | 100 | 100 | 100 | 100 | 100 | 100 | NT |
| CA (%) | 100 | 100 | 100 | 100 | 100 | 100 | 100 | 50 | 100 | 100 | 100 | 100 | 100 | 100 | NT |
| VME | 0 | 0 | 0 | - | - | - | - | - | - | - | - | - | - | - | NT |
| ME | 0 | - | - | 0 | 0 | 0 | 0 | 0 | 0 | 0 | 0 | 0 | 0 | 0 | NT |
| mE | 0 | 0 | 0 | 0 | 0 | 0 | 0 | 1(50%) | 0 | 0 | 0 | 0 | 0 | 0 | NT |
| ***Enterobacter cloacae* complex** (n=2) (one strain was not tested for colistin) | | | | | | | | | | | | | | | |
| EA (%) | 100 | 100 | 100% | 100 | 50 | 100 | 100 | 100 | 100 | 100 | 100 | 100 | 100 | 100 | NT |
| CA (%) | 100 | 100 | 100% | 100 | 50 | 100 | 100 | 100 | 100 | 100 | 100 | 100 | 100 | 100 | NT |
| VME | 0 | 0 | 0 | - | 50 | - | - | - | - | - | - | - | - | - | NT |
| ME | 0 | - | 0 | 0 | 0 | 0 | 0 | 0 | 0 | 0 | 0 | 0 | 0 | 0 | NT |
| mE | 0 | 0 | 0 | 0 | 0 | 0 | 0 | 0 | 0 | 0 | 0 | 0 | 0 | 0 | NT |
| ***Citrobacter freundii*** (n=1) | | | | | | | | | | | | | | | |
| EA (%) | 100 | 100 | 100 | 100 | 100 | 100 |  | 100 | 100 | 100 | 100 | 100 | 100 | 100 | NT |
| CA (%) | 100 | 100 | 100 | 100 | 100 | 100 |  | 100 | 100 | 100 | 100 | 100 | 100 | 100 | NT |
| VME | 0 | 0 | 0 | - | - | - |  | - | - | - | - | - | - | - | NT |
| ME | 0 | - | - | 0 | 0 | 0 |  | 0 | 0 | 0 | 0 | 0 | 0 | 0 | NT |
| mE | 0 | 0 | 0 | 0 | 0 | 0 |  | 0 | 0 | 0 | 0 | 0 | 0 | 0 | NT |
| ***Citrobacter koseri*** (n=1) | | | | | | | | | | | | | | | |
| EA (%) | 0 | NT | 100 | 100 | 100 | 100 | 100 | 100 | 0 | 100 | 100 | 100 | 100 | 100 | NT |
| CA (%) | 100 | NT | 100 | 100 | 100 | 100 | 100 | 100 | 100 | 100 | 100 | 100 | 100 | 100 | NT |
| VME | 0 | NT | 0 | - | - | - | - | - | - | - | - | - | - | - | NT |
| ME | - | NT | - | 0 | 0 | 0 | 0 | 0 | 0 | 0 | 0 | 0 | 0 | 0 | NT |
| mE | 0 | NT | 0 | 0 | 0 | 0 | 0 | 0 | 0 | 0 | 0 | 0 | 0 | 0 | NT |
| ***Providencia stuartii*** (n=1) | | | | | | | | | | | | | | | |
| EA (%) | 0 | 100 | 0 | 100 | 100 | 100 | 100 | 100 | 100 | 0 | 0 | 0 | 100 | NA | NT |
| CA (%) | 100 | 100 | 100 | 100 | 100 | 100 | 100 | 0 | 100 | 0 | 0 | 0 | 100 | NA | NT |
| VME | 0 | - | 0 | - | - | - | - | - | - | 1(100%) | 1(100%) | 1(100%) | - | NA | NT |
| ME | - | 0 | - | 0 | 0 | 0 | 0 | 0 | 0 | - | - | - | 0 | NA | NT |
| mE | 0 | 0 | 0 | 0 | 0 | 0 | 0 | 1(100%) | 0 | 0 | 0 | 0 | 0 | NA | NT |
| ***Proteus mirabilis*** (n=4) | | | | | | | | | | | | | | | |
| EA (%) | 100 | 100 | 100 | 100 | 75 | 75 | 50 | 25 | 0 | 100 | 100 | 100 | 100 | NA | NT |
| CA (%) | 100 | 75 | 100 | 100 | 100 | 75 | 50 | 0 | 75 | 100 | 100 | 100 | 100 | NA | NT |
| VME | 0 | 0 | - | - | 0 | - | 0 | - | - | 0 | 0 | 0 | - | NA | NT |
| ME | 0 | 1(33.3%) | 0 | 0 | 0 | 0 | 1(33.3%) | 3(75%) | 0 | 0 | 0 | 0 | 0 | NA | NT |
| mE | 0 | 0 | 0 | 0 | 0 | 1(25%) | 1(25%) | 1(25%) | 1(25%) | 0 | 0 | 0 | 0 | NA | NT |
| ***Raoultella ornithinolytica*** (n=2) | | | | | | | | | | | | | | | |
| EA (%) | 50 | 100 | 100 | 100 | 100 | 100 | 100 | 100 | 100 | 100 | 100 | 100 | 100 | 100 | NT |
| CA (%) | 100 | 100 | 100 | 100 | 100 | 100 | 100 | 100 | 100 | 100 | 100 | 100 | 100 | 100 | NT |
| VME | 0 | 0 | - | - | - | - | - | - | - | - | - | - | - | - | NT |
| ME | - | - | 0 | 0 | 0 | 0 | 0 | 0 | 0 | 0 | 0 | 0 | 0 | 0 | NT |
| mE | 0 | 0 | 0 | 0 | 0 | 0 | 0 | 0 | 0 | 0 | 0 | 0 | 0 | 0 | NT |
| ***Salmonella* sp.** (n=1) | | | | | | | | | | | | | | | |
| EA (%) | 100 | 100 | 100 | 100 | 100 | 100 | 100 | 100 | 100 | 100 | 100 | NT | 100 | NT | NT |
| CA (%) | 100 | 100 | 100 | 100 | 100 | 100 | 100 | 100 | 100 | 100 | 100 | NT | 100 | NT | NT |
| VME | - | - | - | - | - | - | - | - | - | - | - | NT | - | NT | NT |
| ME | 0 | 0 | 0 | 0 | 0 | 0 | 0 | 0 | 0 | 0 | 0 | NT | 0 | NT | NT |
| mE | 0 | 0 | 0 | 0 | 0 | 0 | 0 | 0 | 0 | 0 | 0 | NT | 0 | NT | NT |
| ***Pseudomonas aeruginosa*** (n=2) | | | | | | | | | | | | | | | |
| EA (%) | NA | NA | NA | 100 | NA | 50 | 100 | 100 | 50 | 100 | 100 | NA | 100 | 100 | NT |
| CA (%) | NA | NA | NA | 100 | NA | 100 | 100 | 100 | 50 | 100 | 100 | NA | 100 | 100 | NT |
| VME | NA | NA | NA | - | NA | 0 | - | - | - | - | - | NA | - | - | NT |
| ME | NA | NA | NA | - | NA | - | - | - | 1(50%) | - | - | NA | 0 | 0 | NT |
| mE | NA | NA | NA | 0 | NA | 0 | 0 | 0 | 0 | 0 | 0 | NA | 0 | 0 | NT |
| ***Stenotrophomonas maltophilia*** (n=1) | | | | | | | | | | | | | | | |
| EA (%) | NA | NA | NA | NA | NA | NA | NA | NA | NA | NA | NA | NA | NA | NA | 100 |
| CA (%) | NA | NA | NA | NA | NA | NA | NA | NA | NA | NA | NA | NA | NA | NA | 100 |
| VME | NA | NA | NA | NA | NA | NA | NA | NA | NA | NA | NA | NA | NA | NA | - |
| ME | NA | NA | NA | NA | NA | NA | NA | NA | NA | NA | NA | NA | NA | NA | - |
| mE | NA | NA | NA | NA | NA | NA | NA | NA | NA | NA | NA | NA | NA | NA | 0 |
| **Total** | | | | | | | | | | | | | | | |
| EA (%) | 86.8 | 86.5 | 95.3 | 96.3 | 93.4 | 92.7 | 93.6 | 96.3 | 92.7 | 95.5 | 96.3 | 92.5 | 94.5 | 98.1 | 100 |
| CA (%) | 93.5 | 84.8 | 83.3 | 94.5 | 93.5 | 90.8 | 93.6 | 92.7 | 97.2 | 94.5 | 93.6 | 92.5 | 94.5 | 99.1 | 100 |
| VME | 7(9.1%) | 9(14.1%) | 3(10.7%) | 3(21.4%) | 4(18.2%) | 2(10%) | 2(10.5%) | 0 | 0 | 3(10%) | 3(10.7%) | 7(41.2%) | 6(100%) | 0 | - |
| ME | 0 | 7(17.1%) | 14(18.9%) | 2(2.2%) | 1(1.2%) | 1(1.2%) | 2(2.3%) | 3(2.9%) | 1(0.9%) | 1(1.3%) | 3(3.8%) | 1(1.1%) | 0 | 1(1%) | - |
| mE | 0 | 0 | 0 | 1(0.9%) | 1(1.9%) | 7(6.4%) | 3(2.8%) | 5(4.6%) | 2(1.8%) | 2(1.8%) | 1(0.9%) | 0 | 0 | 0 | 0 |

AMP: ampicillin, PIP: Piperacillin, AMC: amoxicillin-clavulanate, PTZ: piperacillin-tazobactam, CTX: cefotaxime, CAZ: ceftazidime, CEF: cefepime, IMI: imipenem, MER: meropenem, CIP: ciprofloxacin, LVX: levofloxacin, GEN: gentamicin, AMK: amikacin, COL: colistin, STX: trimethoprim/sulfamethoxazol; VME: Very major error, ME: major error, mE: minor error. NA: Not applicable. NT: Not tested.

“-“indicates VME/ME could not be calculated due to absence of resistant/suceptible isolates

**Supplementary Table 2: Microbiological agreement for Gram positive cocci**

|  | **AMP** | **OXA** | **ERI** | **CLI** | **VAN** | **TEI** | **DAP** | **LNZ** | **GEN** | **LVX** | **RIF** | **TET** | **FUS** |
| --- | --- | --- | --- | --- | --- | --- | --- | --- | --- | --- | --- | --- | --- |
| ***Enterococcus faecalis*** (n=8) | | | | | | | | | | | | | |
| EA (%) | 100 | NA | NA | NA | 100 | 100 | NA | 60 | NA | 80 | NA | NA | NA |
| CA (%) | 100 | NA | NA | NA | 100 | 100 | NA | 80 | NA | 60 | NA | NA | NA |
| VME | - | NA | NA | NA | - | - | NA | - | NA | 0 | NA | NA | NA |
| ME | 0 | NA | NA | NA | 0 | 0 | NA | 1(20%) | NA | 2(50%) | NA | NA | NA |
| mE | 0 | NA | NA | NA | 0 | 0 | NA | 0 | NA | 0 | NA | NA | NA |
| ***Enterococcus faecium*** (n=5) | | | | | | | | | | | | | |
| EA (%) | 100 | NA | NA | NA | 100 | 100 | NA | 62.5 | NA | 100 | NA | NA | NA |
| CA (%) | 100 | NA | NA | NA | 100 | 100 | NA | 75 | NA | 100 | NA | NA | NA |
| VME | 0 | NA | NA | NA | 0 | 0 | NA | 0 | NA | 0 | NA | NA | NA |
| ME | - | NA | NA | NA | 0 | 0 | NA | 2(28.6%) | NA | - | NA | NA | NA |
| mE | 0 | NA | NA | NA | 0 | 0 | NA | 0 | NA | 0 | NA | NA | NA |
| CoNS (n=4) | | | | | | | | | | | | | |
| EA (%) | NA | 75 | 100 | 100 | 100 | 100 | 100 | 100 | 100 | 100 | 100 | 100 | 100 |
| CA (%) | NA | 75 | 100 | 75 | 100 | 100 | 100 | 100 | 100 | 75 | 100 | 100 | 100 |
| VME | NA | 0 | 0 | 0 | - | - | - | - | 0 | 0 | - | - | 0 |
| ME | NA | 1(25%) | 0 | 0 | 0 | 0 | 0 | 0 | 0 | 0 | 0 | 0 | 0 |
| mE | NA | 0 | 0 | 1(25%) | 0 | 0 | 0 | 0 | 0 | 1(25%) | 0 | 0 | 0 |
| Total | | | | | | | | | | | | | |
| EA (%) | 100 | 75 | 100 | 100 | 100 | 100 | 100 | 70.6 | 100 | 94.1 | 100 | 100 | 100 |
| CA (%) | 100 | 75 | 100 | 75 | 100 | 100 | 100 | 82.4 | 100 | 82.4 | 100 | 100 | 100 |
| VME | 0 | 0 | 0 | 0 | 0 | 0 | - | 0 | 0 | 0 | - | - | 0 |
| ME | 0 | 1(100%) | 0 | 0 | 0 | 0 | 0 | 3(18.8) | 0 | 2(40%) | 0 | 0 | 0 |
| mE | 0 | 0 | 0 | 1(25%) | 0 | 0 | 0 | 0 | 0 | 1(5.9%) | 0 | 0 | 0 |

AMP: ampicillin, OXA: oxacillin, ERI: erythromycin, CLI: clindamycin, VAN: vancomycin, TEI: teicoplanin, DAP: daptomycin, LZD: linezolid, GEN: gentamicin, LVX: levofloxacin, RIF: rifampin, TET: tetracycline; VME: Very major error, ME: major error, mE: minor error. NA: Not applicable. NT: Not tested. CoNS coagulase-negative *Staphylococcus* spp.

“-“indicates VME/ME could not be calculated due to absence of resistant/suceptible isolates
